# Supplementary figures and images for: A cross-sectional analysis of clinicopathologic similarities and differences between Henoch-Schönlein purpura nephritis and IgA nephropathy
Source: PLoS One. 2020 Apr 23;15(4):e0232194. doi: 10.1371/journal.pone.0232194 (PMC7179927; doi:10.1371/journal.pone.0232194)

A.

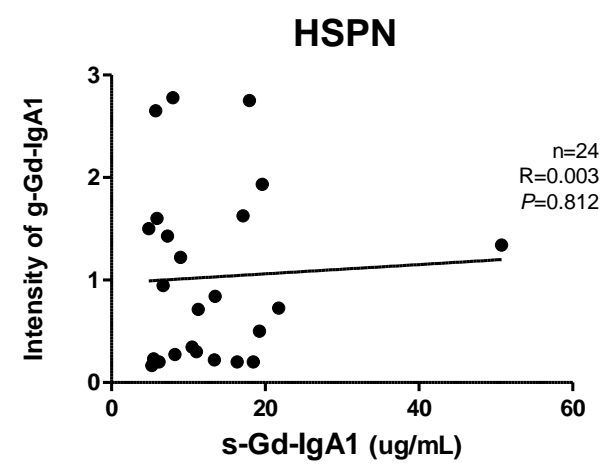

B.

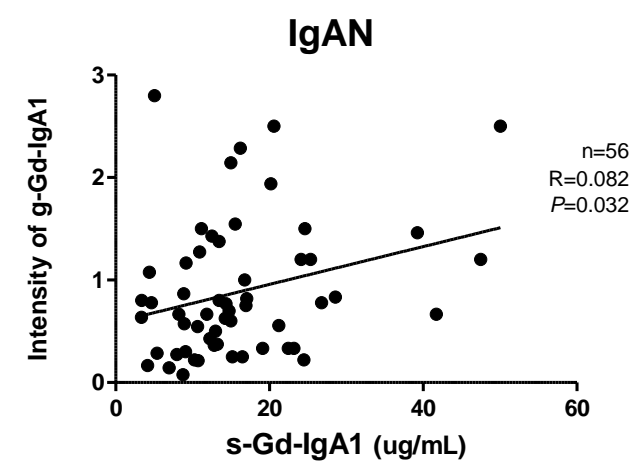

Supplement: S1 Fig — Scatter plots of correlations between g-Gd-IgA1 positivity and s-Gd-IgA1 levels in patients with HSPN (A) and IgAN (B). Data were statistically analyzed using Spearman correlations. (PDF) [file pone.0232194.s001.pdf]

**A.**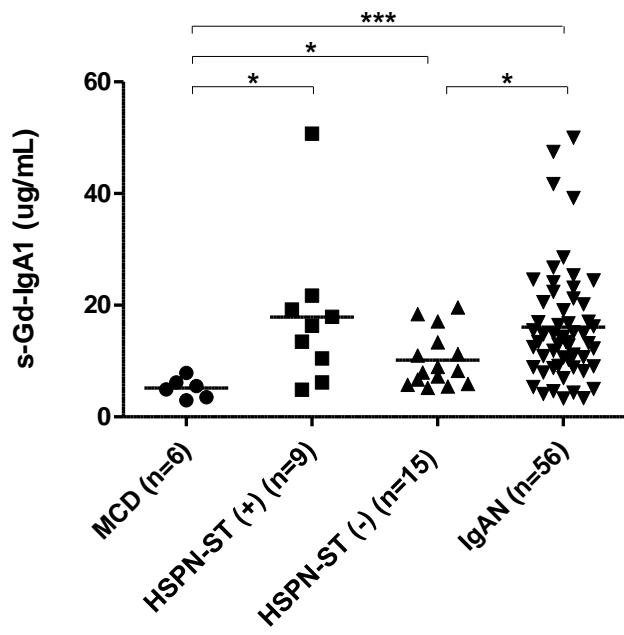**B.**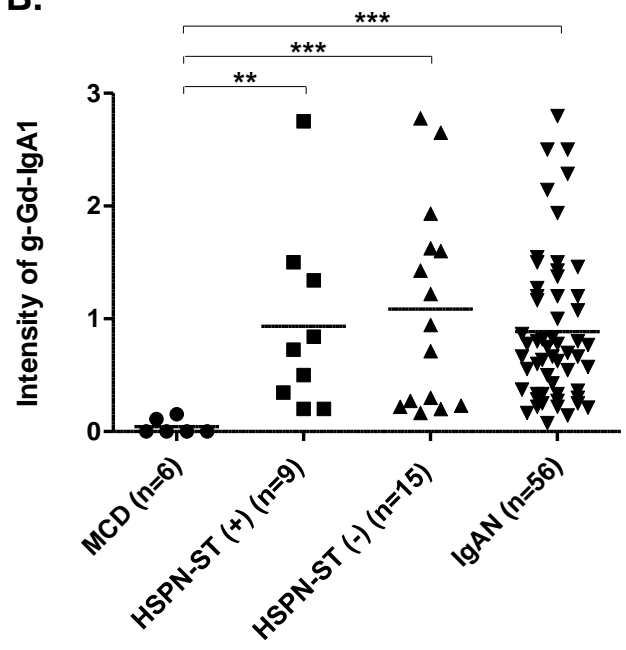**C.**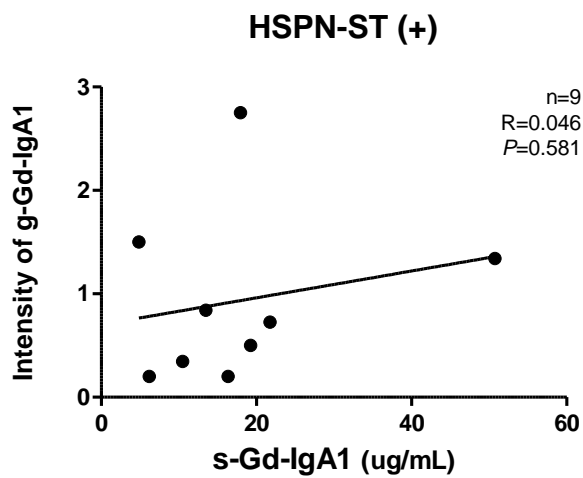**D.**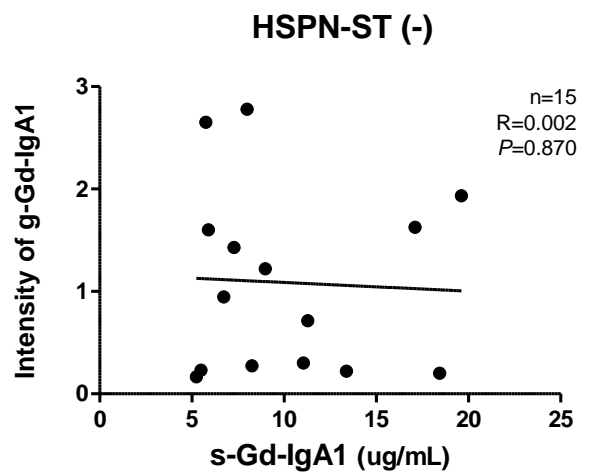

Supplement: S2 Fig — Comparisons of s-Gd-IgA1 levels (A) and g-Gd-IgA1 positivity (B) among MCD patients, HSPN patients who received steroid therapy [HSPN-ST (+)], HSPN patients who did not receive steroid therapy [HSPN-ST (-)], and IgAN patients. Horizontal solid lines represent means. Data were statistically analyzed using Kruskal-Wallis tests and Mann-Whitney U tests. *P<0.05, **P<0.01, and ***P<0.001. Scatter plots of correlations between g-Gd-IgA1 positivity and s-Gd-IgA1 levels in HSPN-ST (+) (C) and HSPN-ST (-) (D). Data were statistically analyzed using Spearman correlations. (PDF) [file pone.0232194.s002.pdf]

**A.**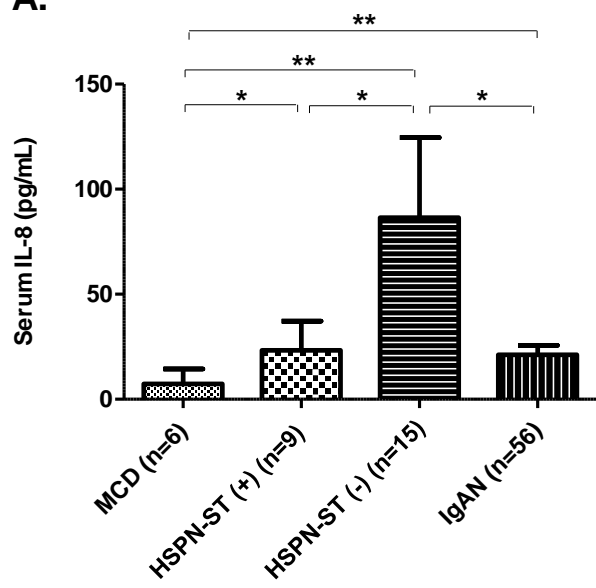**B.**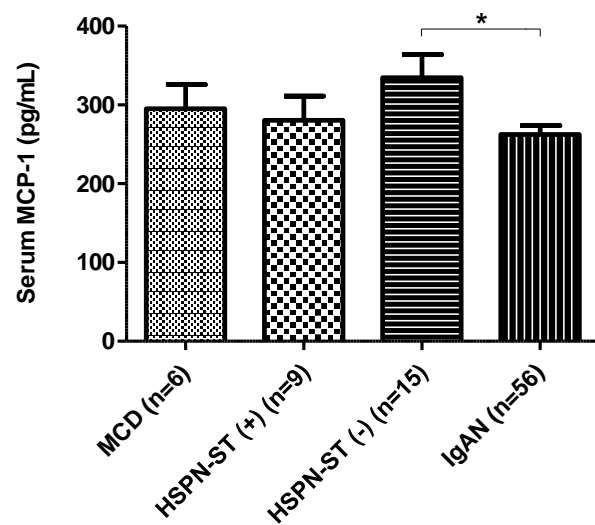**C.**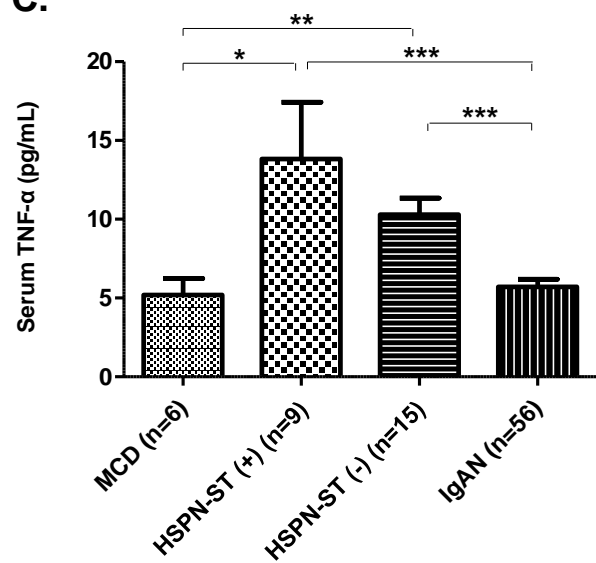**D.**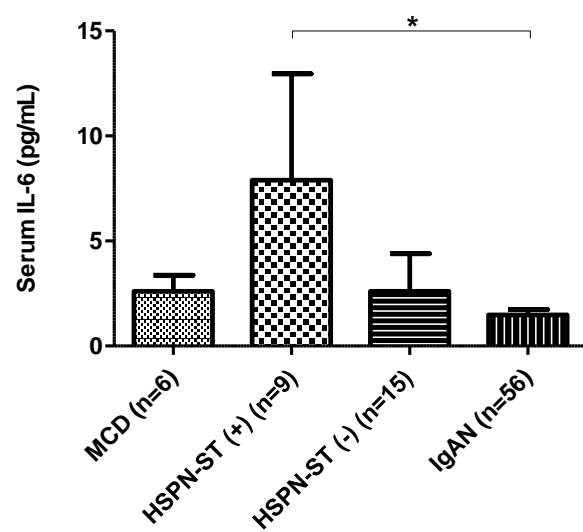

Supplement: S3 Fig — Comparison of serum IL-8 (A), MCP-1 (B), TNF-α (C), and IL-6 (D) levels among MCD patients, HSPN patients who received steroid therapy [HSPN-ST (+)], HSPN patients who did not receive steroid therapy [HSPN-ST (-)], and IgAN patients. Values are presented as means ± SEM. Data were statistically analyzed using Kruskal-Wallis tests and Mann-Whitney U tests. *P<0.05, **P<0.01, and ***P<0.001. (PDF) [file pone.0232194.s003.pdf]

**A.**

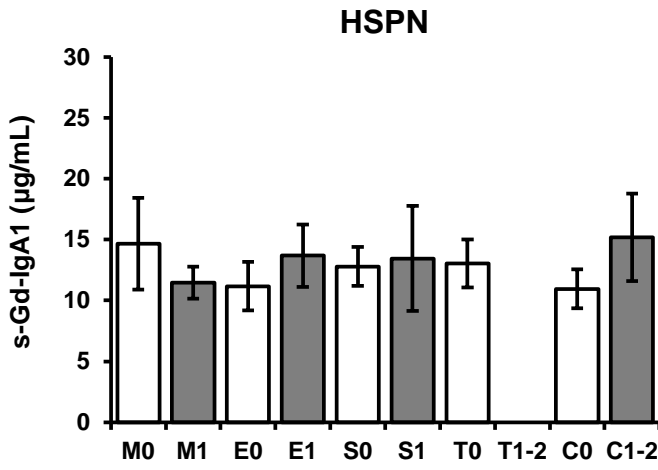

**B.**

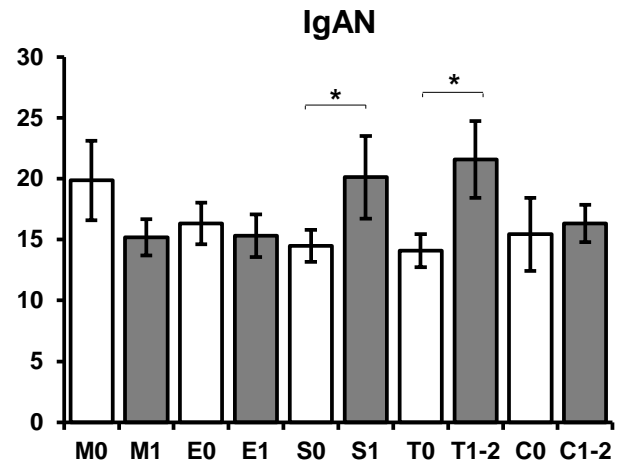

**C.**

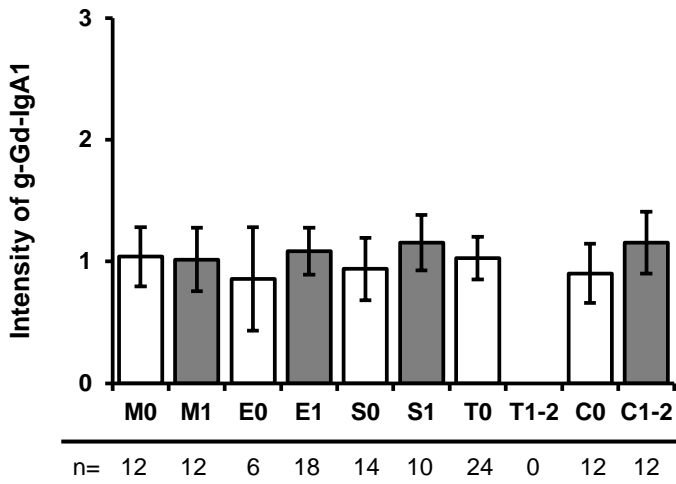

**D.**

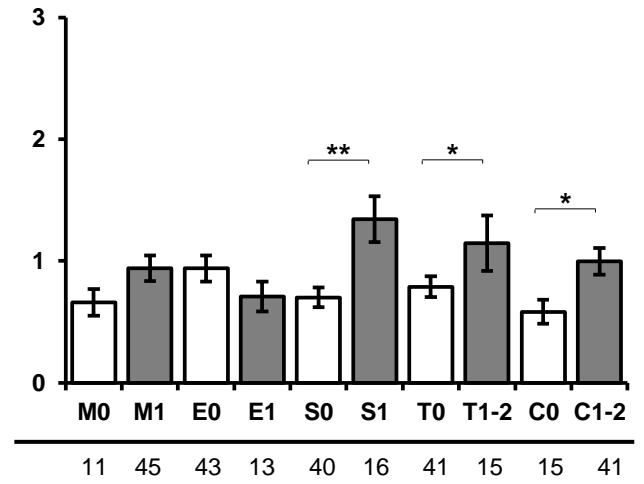

Supplement: S4 Fig — Patients with HSPN (A and C) or IgAN (B and D) were assigned to groups according to mesangial hypercellularity, endocapillary hypercellularity, segmental glomerulosclerosis, and tubular atrophy/interstitial fibrosis. Values are presented as means ± SEM. Data were statistically analyzed using Mann-Whitney U tests. *P<0.05 and **P<0.01. (PDF) [file pone.0232194.s004.pdf]

HSPN only vs. HSPN with arthritis or abdominal pain

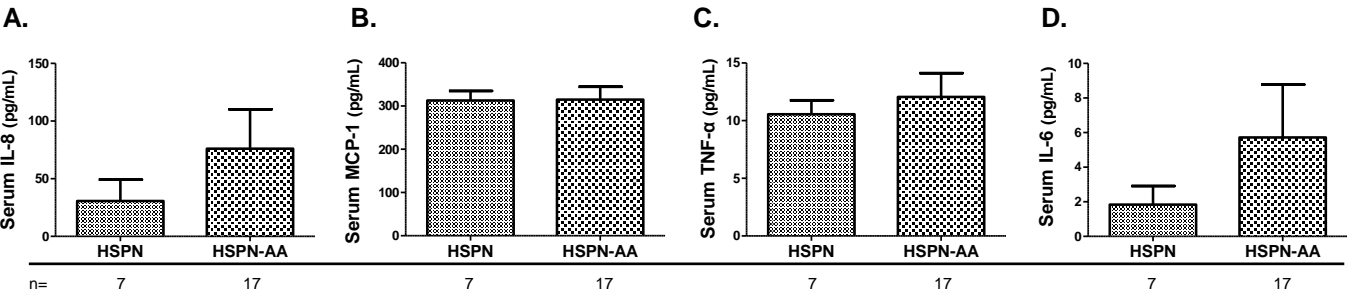

Supplement: S5 Fig — Comparison of serum IL-8 (A), MCP-1 (B), TNF-α (C), and IL-6 (D) levels between patients with HSPN without any systemic symptoms other than nephritis and patients with HSPN with arthritis or abdominal symptoms (HSPN-AA). Values are presented as means ± SEM. Data were statistically analyzed using Mann-Whitney U tests. (PDF) [file pone.0232194.s005.pdf]

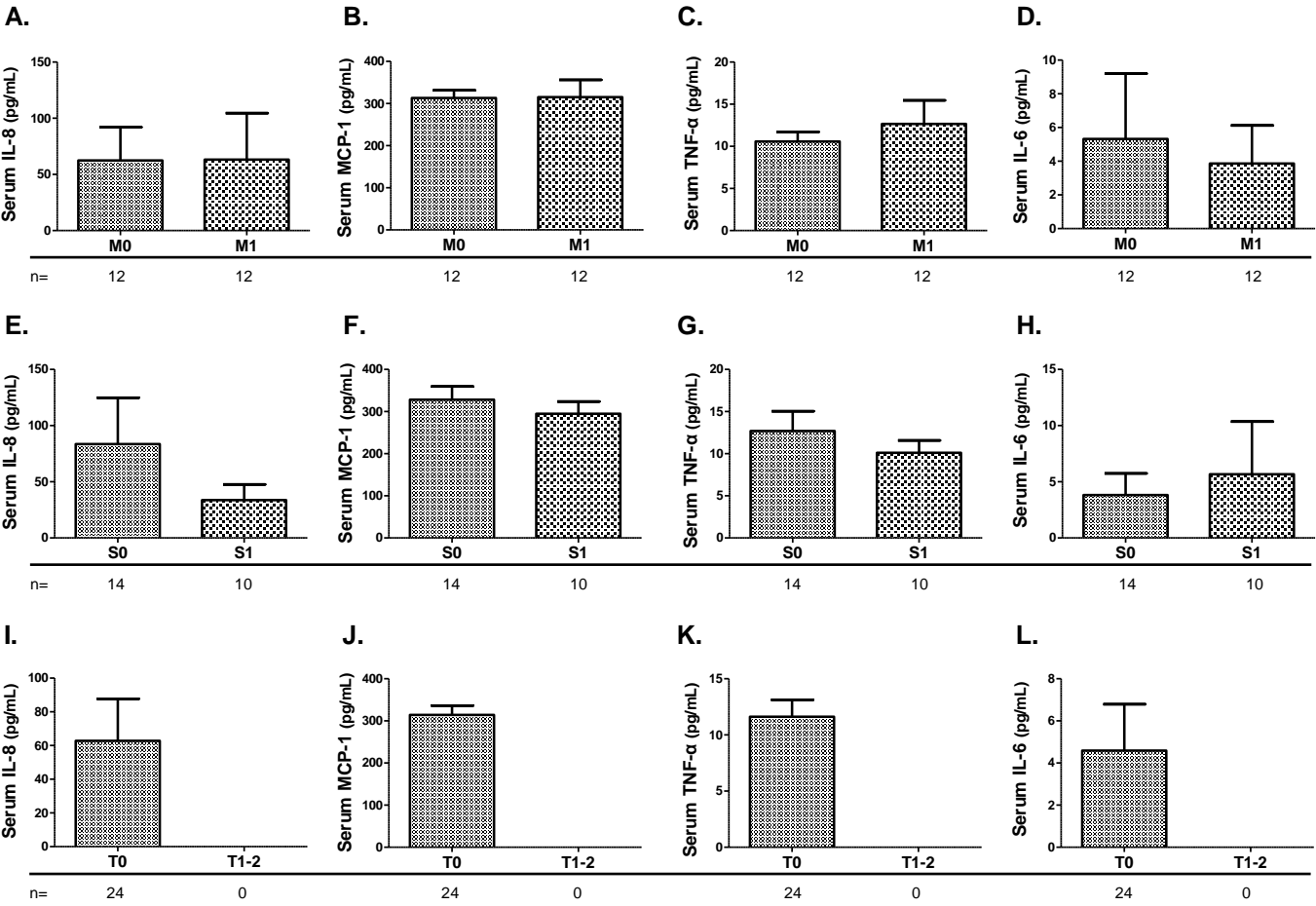

Supplement: S6 Fig — Comparison of serum IL-8 (A, E and I), MCP-1 (B, F and J), TNF-α (C, G and K), and IL-6 (D, H and L) in patients with HSPN according to the presence of mesangial hypercellularity, segmental glomerulosclerosis, and tubular atrophy/interstitial fibrosis based on the Oxford classification. Values are presented as means ± SEM. Data were statistically analyzed using Mann-Whitney U tests. (PDF) [file pone.0232194.s006.pdf]
