# Supplementary material for: A cross-sectional analysis of clinicopathologic similarities and differences between Henoch-Schönlein purpura nephritis and IgA nephropathy
Source: PLoS One. 2020 Apr 23;15(4):e0232194. doi: 10.1371/journal.pone.0232194 (PMC7179927; doi:10.1371/journal.pone.0232194)
Supplement: S1 Table — (RTF) [file pone.0232194.s007.rtf]

S1 Table. Correlation between both types of Gd-IgA1 and inflammatory cytokines in HSPN patients with or without steroid therapy at the time of renal biopsy. 
�@	HSPN-ST (+) (n=9)	HSPN-ST (-) (n=15)	
�@	s-Gd-IgA1 (ìg/mL)	g-Gd-IgA1 intensity	 s-Gd-IgA1 (ìg/mL)	g-Gd-IgA1 intensity	
Variable	R value 	P value	R value 	P value 	R value 	P value	R value 	P value 	
IL-8 (pg/mL)	0.175	0.262	0.047	0.574	0.087	0.287	0.209	0.087	
MCP-1 (pg/mL)	0.014	0.759	0.091	0.431	0.002	0.874	0.245	0.061	
TNF-á (pg/mL)	0.054	0.547	0.134	0.332	0.056	0.394	0.247	0.059	
IL-6 (pg/mL)	0.826	<0.001	0.022	0.702	0.018	0.634	0.268	0.048	

Data were statistically analyzed using Spearman correlation tests. 
Abbreviations: HSPN, Henoch-Schönlein purpura nephritis; IgAN, Immunoglobulin A nephropathy; ST, steroid therapy.
