# Supplementary material for: A cross-sectional analysis of clinicopathologic similarities and differences between Henoch-Schönlein purpura nephritis and IgA nephropathy
Source: PLoS One. 2020 Apr 23;15(4):e0232194. doi: 10.1371/journal.pone.0232194 (PMC7179927; doi:10.1371/journal.pone.0232194)
Supplement: Attachment — Submitted filename: Response to Reviewer.docx [file pone.0232194.s010.docx]

April 5, 2020

Dr. Fabio Sallustio

Academic Editor

*PLOS ONE*

RE: PONE-D-20-00737R1

A cross-sectional analysis of clinicopathologic similarities and differences between Henoch-Schönlein purpura nephritis and IgA nephropathy

Dear Dr. Sallustio:

We really appreciate the meticulous review and are pleased to learn that our submission is of interest. We appreciate the opportunity to submit a revised manuscript. All changes, including minor modifications, are shown in red in the revised manuscript using track changes.

We have addressed the concerns raised as follows:

**Journal requirements**

1. If you would like to make changes to your financial disclosure, please include your updated statement in your cover letter.

No changes are required regarding our financial disclosure.

2. To enhance the reproducibility of your results, we recommend that if applicable you deposit your laboratory protocols in protocols.io, where a protocol can be assigned its own identifier (DOI) such that it can be cited independently in the future.

Because the experimental protocols are clearly detailed in the materials and methods section, we did not prepare a separate specific laboratory protocol.

**Reviewer 1**

After these corrections the revised paper will be suitable for publication.

1. Page 26 line 16 delete were

Thank you so much for your suggestion. We deleted “were” in line 16 of Page 26.

2. Pag 26 line 18 these results let us to presume

As you pointed out, that sentence appeared to be inappropriate. We modified as follow; These results led us to presume that elevated levels of serum inflammatory cytokines in HSPN may be related to the formation of Gd-IgA1 and the progression of nephritis.

**Reviewer 2**

Recommendation: Accept With No Changes

The authors have completely addressed all the issues raised by reviewers

We really appreciate your careful review of our manuscript. We completed to modify some inappropriate parts of the manuscript.

We feel that we have addressed all of the concerns of the referees and that the manuscript has been significantly improved. We hope that you find the revised version of our manuscript suitable for publication in *PLOS ONE.*

We look forward to receiving your response.

Sincerely,

Yukihiro Wada

Division of Nephrology, Department of Medicine

Showa University School of Medicine

Tokyo, Japan

Address: 1-5-8 Hatanodai, Shinagawa-ku, Tokyo 142-8666, Japan

Tel: +81-3-3784-8533

E-mail address: yukihiro@med.showa-u.ac.jp
